# Molecular dynamics of the human RhD and RhAG blood group proteins

**DOI:** 10.3389/fchem.2024.1360392

**Published:** 2024-03-19

**Authors:** Aline Floch, Tatiana Galochkina, France Pirenne, Christophe Tournamille, Alexandre G. de Brevern

**Affiliations:** ^1^ University Paris Est Créteil, INSERM U955 Equipe Transfusion et Maladies du Globule Rouge, IMRB, Créteil, France; ^2^ Laboratoire de Biologie Médicale de Référence en Immuno-Hématologie Moléculaire, Etablissement Français du Sang Ile-de-France, Créteil, France; ^3^ Université Paris Cité and Université des Antilles and Université de la Réunion, Biologie Intégrée du Globule Rouge, UMR_S1134, BIGR, INSERM, DSIMB Bioinformatics team, Paris, France

**Keywords:** Rh blood group system, membrane proteins, molecular models, molecular dynamics simulation, protein structural elements, structural alphabet

## Abstract

**Introduction:** Blood group antigens of the RH system (formerly known as “Rhesus”) play an important role in transfusion medicine because of the severe haemolytic consequences of antibodies to these antigens. No crystal structure is available for RhD proteins with its partner RhAG, and the precise stoichiometry of the trimer complex remains unknown.

**Methods:** To analyse their structural properties, the trimers formed by RhD and/or RhAG subunits were generated by protein modelling and molecular dynamics simulations were performed.

**Results:** No major differences in structural behaviour were found between trimers of different compositions. The conformation of the subunits is relatively constant during molecular dynamics simulations, except for three large disordered loops.

**Discussion:** This work makes it possible to propose a reasonable stoichiometry and demonstrates the potential of studying the structural behaviour of these proteins to investigate the hundreds of genetic variants relevant to transfusion medicine.

## 1 Introduction

In transfusion medicine, blood group antigens from the RH system (formerly known as « Rhesus ») play a crucial role (Chou and Westhoff, 2010; Thornton and Grimsley, 2019). Firstly, because of their immunogenicity (ability to induce antibody formation in recipients) and secondly, because of the severe haemolytic consequences antibodies to these antigens can induce: post-transfusion haemolysis, haemolytic disease of the foetus and the newborn, transfusion impasse (Noizat-Pirenne, 2012; de Haas et al., 2015). Antibody formation may occur when an individual is exposed to foreign antigens through transfusion or pregnancy, but also depends on mostly unknown individual factors (Tormey and Hendrickson, 2019).

RH antigens are expressed on the red blood cell (RBC) membrane by two Rh proteins, i.e., RhD and RhCE. The proteins result from the expression of the highly homologous genes *RHD* and *RHCE*, situated on chromosome 1, at locus 1p36.11. They are associated with 5 main antigens: D, C, E, c and e (also named RH1, RH2, RH3, RH4 and RH5, respectively). The *RHD* gene, when present, produces the RhD protein, which expresses the D antigen. The *RHCE* gene produces the RhCE protein with 4 common alleles resulting in the expression of the C, c, E and e antigens (each allele combines the expression of 2 antigens: C or c associated with E or e). Fifty other antigens have been defined in this blood group system, resulting from altered Rh proteins arising from point mutations, from hybrid alleles (through recombination events between *RHD* and *RHCE*), or from both (Chou and Westhoff, 2010).

RH antigens and phenotypes resulting from novel alleles can be characterized using experimental (serological data: testing with polyclonal antibodies resulting from alloimmunisation, monoclonal antibodies specific of certain epitopes, by flow cytometry … ) and clinical data (if at least one carrier has produced antibodies to the standard antigen or if no antibodies have been detected despite many carriers having been exposed) (Chou and Westhoff, 2010). These methods provide only indirect information about the protein conformation and behaviour, and assumptions based on them are unreliable. Indeed, while several alleles were initially thought to present no alloimmunisation risk, individuals homozygous for these alleles have produced alloantibodies to the standard antigen, revealing that the antigen was, in fact, altered (St-Louis et al., 2011; Pham et al., 2013). Moreover, experimental data is unavailable for many alleles, especially the rare ones. In those cases, the assessment depends solely on whether the amino acid substitutions are exposed at the extracellular RBC membrane. Such a position is considered to surely alter the antigen’s epitopes. We have proposed the most complete database dedicated to Rh variants. This dedicated database is named RHeference and contains entries for 710 *RHD* alleles, 11 *RHCE* alleles, 30 phenotype descriptions, it also covers also partly characterized alleles, haplotypes, and some miscellaneous entries, with molecular, phenotypic, serological, alloimmunization, haplotype, geographical, and other data, detailed for each source (Floch et al., 2021a).

Molecular modelling techniques have the potential to provide new and more direct information on Rh protein structure and dynamics (Burton and Daniels, 2011; de Brevern et al., 2018; Floch et al., 2021b), which is crucially important to better understand the expression of RH antigens and antibody formation in transfusion medicine. The first two major questions that could be addressed using molecular models are the three-dimensional structure of RhD monomers, and the structure and stability of RhD/RhAG trimers of different compositions. The monomer structure can be modelled by homology, i.e., mapping protein sequence to the structure of a resolved protein homolog used as a template. The composition and the stability of the trimers are more complex questions that can be addressed by running molecular dynamics (MD) simulations to reproduce protein movements at the atomistic level.

The main challenge in the development of reliable models of Rh/RhAG trimers for molecular dynamics is the choice of the structural template and identification of the trimer arrangement to be used as the initial conformation for the simulations. The Rh proteins RhD and RhCE and their main partner, RhAG (“Rhesus Associated Glycoprotein”) (Chou and Westhoff, 2010), are highly homologous proteins, with 12 membrane-spanning domains. They are part of the Amt/MEP/Rh superfamily of ammonium transporters, with a trimeric structure (Khademi et al., 2004; Li et al., 2007; Lupo et al., 2007; Gruswitz et al., 2010). No crystal structure has been published for the human Rh blood group proteins at the time when this project was initiated, but the structure of the human RhCG protein (PDB ID: 3HD6) (Gruswitz et al., 2010) and of the bacterial homolog NeRh50 from *Nitrosomonas europaea* (PDB ID: 3B9W) (Li et al., 2007; Lupo et al., 2007) have been resolved. RhCG shares 31.65% sequence identity with RhD, and 52.32% with RhAG, while NeRh50 has 24.86% sequence identity with RhD, and 35.75% with RhAG. Experimental data suggests that RhD and RhCE monomers are not associated in the same trimer (Avent et al., 1988), and that the composition of the human Rh trimer is likely to be 2 RhAG monomers for 1 RhD or RhCE monomer (Mouro-Chanteloup et al., 2002; Floch et al., 2021b). Homotrimers of RhAG can also be detected experimentally, when the *RHD* and *RHCE* genes are both altered. In contrast, when the *RHAG* gene is altered, no RhD or RhCE proteins are expressed (Chou and Westhoff, 2010), which reveals that RhD_3_ and RhCE_3_ homotrimers do not exist. The 37th residue of RhAG is glycosylated but neither RhD nor RhCE are glycosylated (Anstee and Tanner, 1993).

A few 3D models for the study of Rh proteins have been proposed previously by homology modelling. These propositions have been based on bacterial homologues, and/or on automated modelling methods (Khademi et al., 2004; Conroy et al., 2005; Callebaut et al., 2006; Lupo et al., 2007; Flegel et al., 2008; Tilley et al., 2010; Silvy et al., 2012). Our team has recently proposed, first a new multi-template model of the RhD monomer (de Brevern et al., 2018) based on the human RhCG and NeRh50 as templates, followed by a model of the RhD-RhAG trimer (Floch et al., 2021b), based on the human RhCG (the multi-template approach did not lead to better models), using state-of-the-art methods specific for transmembrane proteins. A recent electron microscopy structure was made available for RhCE in complex with RhAG and ankyrine [PDB id 7uzq (Vallese et al., 2022)] and used to propose monomer models (Trueba-Gómez et al., 2023). A few teams have modelled the trimer that Rh proteins form with RhAG, but none have performed molecular dynamics (MD) simulations for these proteins. This study focuses on the trimer(s) formed by RhD and RhAG proteins. We propose the best RhAG_3_ and RhAG_2_RhD models by homology modelling and study the molecular dynamics behaviour of the proteins through MD simulations. In recent years, deep learning approaches have had a major impact on the development of 3D protein models (Jumper et al., 2021), so we will also explore the potential benefits of AlphaFold2, for which some limitations have been also underlined (He et al., 2023; Tourlet et al., 2023).

## 2 Materials and methods

### 2.1 Comparative modelling of RhAG/RhD trimers

Since no structure for the human Rh proteins (RhD, UniProt id Q02161 and RhCE, UniProt id P18577) (Consortium, 2015) had been resolved at the time of our study, Rh trimers were modelled by homology (de Brevern, 2010) as previously published for the RhD monomer (de Brevern et al., 2018). Sequence’s conservation was assessed with Consurf server (Ashkenazy et al., 2016), then homologous protein sequences for which protein structures are available were found by a query of the sequences of the target proteins (here, RhAG and RhD) with PSI-BLAST (Altschul et al., 1997) and HHpred (Hildebrand et al., 2009), using the Protein DataBank (PDB) database (Berman et al., 2002). The selected structures were analysed with MolProbity (Chen et al., 2010) and ProCheck (Laskowski et al., 1993). The sequences were carefully aligned with Clustal Omega (Sievers et al., 2011). Protein structures and/or structural models were superimposed using the McLachlan algorithm (McLachlan, 1982) as implemented in the program ProFit 3.1, and using iPBA (Gelly et al., 2011), and TM-Align (Zhang and Skolnick, 2005). Secondary structure was predicted by several specific methods, such as HMMTOP (Tusnády and Simon, 2001) and MEMSAT (Jones, 2007). Hundreds of structural models were generated with Modeller 9.12 (Sali and Blundell, 1993; Martí-Renom et al., 2000). These models were analysed and compared with TM-Align (Zhang and Skolnick, 2005), the Discrete Optimized Protein Energy (DOPE) (Shen and Sali, 2006) potential implemented in Modeller, the Hybrid Protein Model (HPM, dedicated to transmembrane proteins) (de Brevern and Hazout, 2003; Esque et al., 2015; Téletchéa et al., 2023) webserver and the MAIDEN approach (Postic et al., 2016a). The positioning of the models within the lipid bilayer were performed by OPM (Lomize et al., 2006; Lomize et al., 2011) and OREMPRO (Postic et al., 2016b). The best structural model was selected.

**FIGURE 1:**
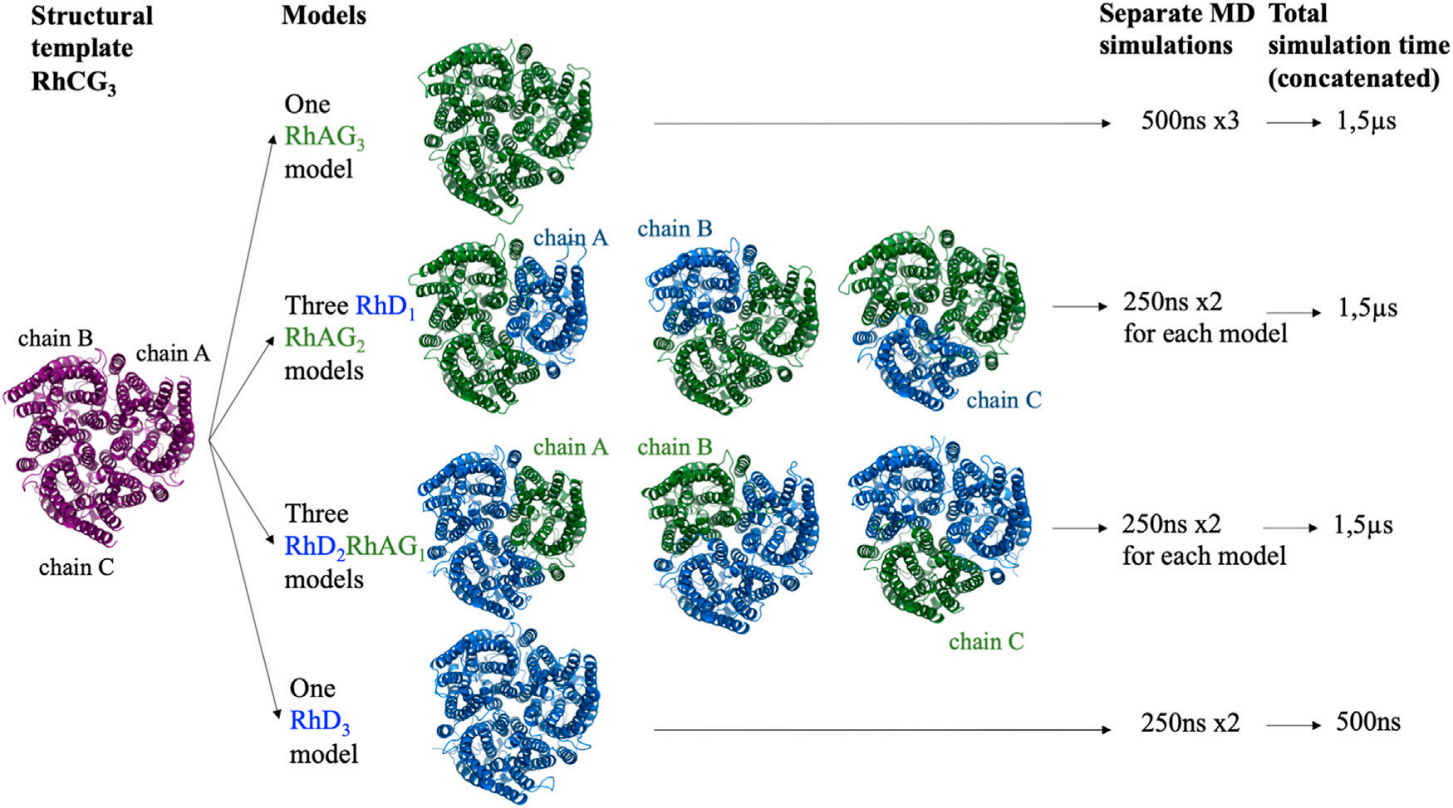
Schematic representation of all the complexes of RhD and RhAG proteins modelled and for which molecular dynamics simulations were performed. Purple: RhCG, the structural template. Blue: RhD subunits. Green: RhAG subunits.

Using a symmetrical homotrimer of human RhCG (PDB ID: 3hd6), whose multimeric state was built with PDBePISA (Proteins, Interfaces, Structures and Assemblies) (Krissinel and Henrick, 2007), trimers of all the potential compositions were modelled: one RhAG_3_ homo-trimer, three RhD_1_RhAG_2_ trimers (with the RhD monomer positioned either as chain A, B or C), three RhD_2_RhAG_1_ (with the RhAG monomer positioned either as chain A, B or C) and one RhD_3_ homo-trimer (Figure 1). RhCG (PDB id 3hd6) was used as a structural template. The recent structure of RhCE in complex with RhAG and ankyrine [PDB id 7uzq (Vallese et al., 2022)] and AlphaFold2 model (Jumper et al., 2021) will be discussed in Discussion section.

### 2.2 Molecular dynamics simulations protocol

The membrane systems were prepared using the CHARMM-GUI webserver (Wu et al., 2014). Each trimer was inserted into a rectangular box, with a palmitoyl-oleoyl phosphatidyl choline (POPC) bilayer, solvated with TIP3P water model (Jorgensen et al., 1983) and neutralized with Na^+^ and Cl^−^ counter ions at a concentration of 0.15 M. The molecular dynamics (MD) simulations were performed with GROMACS 2016.4 (Van Der Spoel et al., 2005; Abraham et al., 2015) using all-atom CHARMM36 forcefield (Huang et al., 2013).

The system geometry was optimized by minimizing the energy with a steepest-descent algorithm for 5,000 steps, keeping the positions of the heavy atoms of the proteins fixed. As recommended by the CHARMM-GUI webserver output (Lee et al., 2000), the system was then equilibrated in six short runs from 25 ps to 100 ps each, using the Berendsen algorithm for temperature and pressure control (Berendsen et al., 1984), with time coupling constraints of 0.1 ps, and gradually releasing constraints keeping the positions of the heavy atoms of the proteins fixed. The resulting equilibrated systems were used as an initial condition for the production runs of 250–500 ns with all the constraints removed.

The MD production runs were performed with the temperature fixed at 310,15 K (to approximate body temperature). Separate temperature coupling was applied for the protein, lipids and solvent molecules using the Nose-Hoover algorithm (Nosé, 1984; Hoover, 1985), with a coupling constant of 1.0 ps. The MD runs were performed with the pressure fixed at 1 bar, with semi-isotropic coupling using the Parrinello-Rahman barostat (Parrinello and Rahman, 1981) with a coupling constant of 5.0 ps. Periodic boundary conditions were used. Long-range electrostatic interactions were handled with the Particle Mesh Ewald (PME) algorithm (Darden et al., 1999). The lengths of the covalent bonds between hydrogen and heavy atoms were fixed using the LINCS algorithm (Hess et al., 1997), which enabled the use of an integration step of 2 fs.

Three independent 500 ns runs were performed for the RhAG_3_ system, for a cumulative length of 1.5 µs? Two independent 250 ns runs were performed for each of the other systems (see Figure 1), for a cumulative length of 1.5µs for RhD_1_RhAG_2_ systems (regardless of the chain which was RhD), 1.5µs for RhD_2_RhAG_1_ systems (regardless of the chain which was RhAG), and only 500ns for the RhD_3_ system, since the existence of the latter system *in vivo* is unlikely, as explained above.

### 2.3 MD simulation analysis

For the 8 trimer models considered, we obtained MD trajectories with conformations saved every 100 ps. The GROMACS software was used for the analyses of MD simulation trajectories, with in-house Python and R scripts. Root mean square deviations (RMSD) and root mean square fluctuations (RMSF) were computed on Cα atoms. We computed normalized RMSFs for each chain (Bornot et al., 2011).

To analyse local conformations, we used a structural alphabet composed of 16 local prototypes, called Protein Blocks (PBs) (de Brevern et al., 2000; Léonard et al., 2014), to describe the protein backbone conformation based on the dihedral angles of 5 consecutive residues. PBs are labelled from *a* to *p*. PBs *a* to *j* are specific to coils. PBs primarily representing β-strands are: PB *d* which can be roughly described as the prototype for central β-strand; PBs *a* to *c* which represent β-strand N-caps, and PBs *e* and *f* which represent β-strand C-caps. PBs primarily representing α-helices are: PB *m*, which can be roughly described as the prototype for α-helix, PBs *k* and *l* which are specific to α-helix N-caps and PBs *n* to *p* which are specific to α-helix C-caps. PB assignment was carried out for every residue obtained from every snapshot extracted from MD simulations using the PBxplore tool (Barnoud et al., 2017). The flexibility of each position was quantified with the *N*_*eq*_ (for equivalent number of PBs), a statistical measurement similar to entropy. It represents the average number of PBs a residue adopts at a given position (de Brevern et al., 2000).

$$N_{eq}=\exp\left(-\sum_{x=1}^{16}f_{x\;}{\ln\;f}_{x\;}\right)$$

*N*_*eq*_ is calculated as shown here, where, *f*_*x*_ is the frequency of PB *x* at the position of interest. An *N*_*eq*_ value of 1 indicates that only one type of PB is observed, while a value of 16 is equivalent to an equal probability for each of the 16 states, *i.e.*, a random distribution. An *N*_*eq*_ of 1.0 is, by definition, a rigid region of a protein. We have determined that disordered regions have an *N*_*eq*_ of 8.0 or more, while flexible regions have an *N*_*eq*_ around 4.0 (Akhila et al., 2020).

The analysis of conformational variations in terms of PBs and normalized RMSF was performed on the concatenated trajectories. Monomers coming from trimers of identical composition were analysed together (e.g., all trajectories for the RhD subunit within trimers composed of 2 RhAG and 1 RhD subunits).

To compare the local conformational variability between two different trajectories of the same protein, we calculated the total difference between PB frequencies (ΔPB).

$$\Delta PB=\sum_{x=1}^{16}\left|{f_{x}}^{1}-{f_{x}}^{2}\right|$$

To compare the local conformational variability between two different trajectories of the same protein, ΔPB**,** the total difference between PB frequencies, was computed using this formula, where *f*_*x*_^*1*^ *and f*_*x*_^*2*^ are frequencies of PB *x,* in the first and the second trajectory respectively for the same position. The 50 ns at the beginning of each trajectory were truncated to compute average PB and RMSF only at the steady state (once the RMSD reached a plateau).

## 3 Results

### 3.1 Structure of RhAG/RhD model trimers

At the time of our study, out of the potential structural templates identified, human RhCG (PDB id 3hd6) (Gruswitz et al., 2010) was the best template, with the highest sequence identity to both with RhD (31.65%), and with RhAG (52.32%, see Supplementary Table S1). The most conserved part of the sequence corresponds to the transmembrane regions, which is consistent with the evolutionary conservation assessed by Consurf (Ashkenazy et al., 2016) (see Supplementary Figures S1, S2). The target sequence of each trimer formed by RhD and/or RhAG subunits in all compositions was aligned to the sequences of both templates using Clustal Omega (Sievers et al., 2011).

Proposing trimer structure models is challenging. However, the difference in the models generated in our case seemed to be very small. For instance, for the RhAG_3_ trimers composed of a total of 1,227 residues, the average RMSD of the models was 1.56 ± 0.16 Å and ranged from 0.97 to 2.12 Å for the whole trimer, with a minimum coverage of 1,202 residues, as measured by TM-Align (Zhang and Skolnick, 2005). The results were similar between the different models with the same composition (Figure 1). The RMSD for RhD_1_RhAG_2_ trimer models (1,235 residues) was 1.17 ± 0.23 Å and ranged from 0.61 to 1.93 Å, with a minimum coverage of 1,195 residues, and the RMSD for RhD_2_RhAG_1_ trimer models (1,243 residues) was 1.03 ± 0.24 Å and ranged from 0.54 to 1.73 Å, with a minimum coverage of 1,208 residues.

**FIGURE 2:**
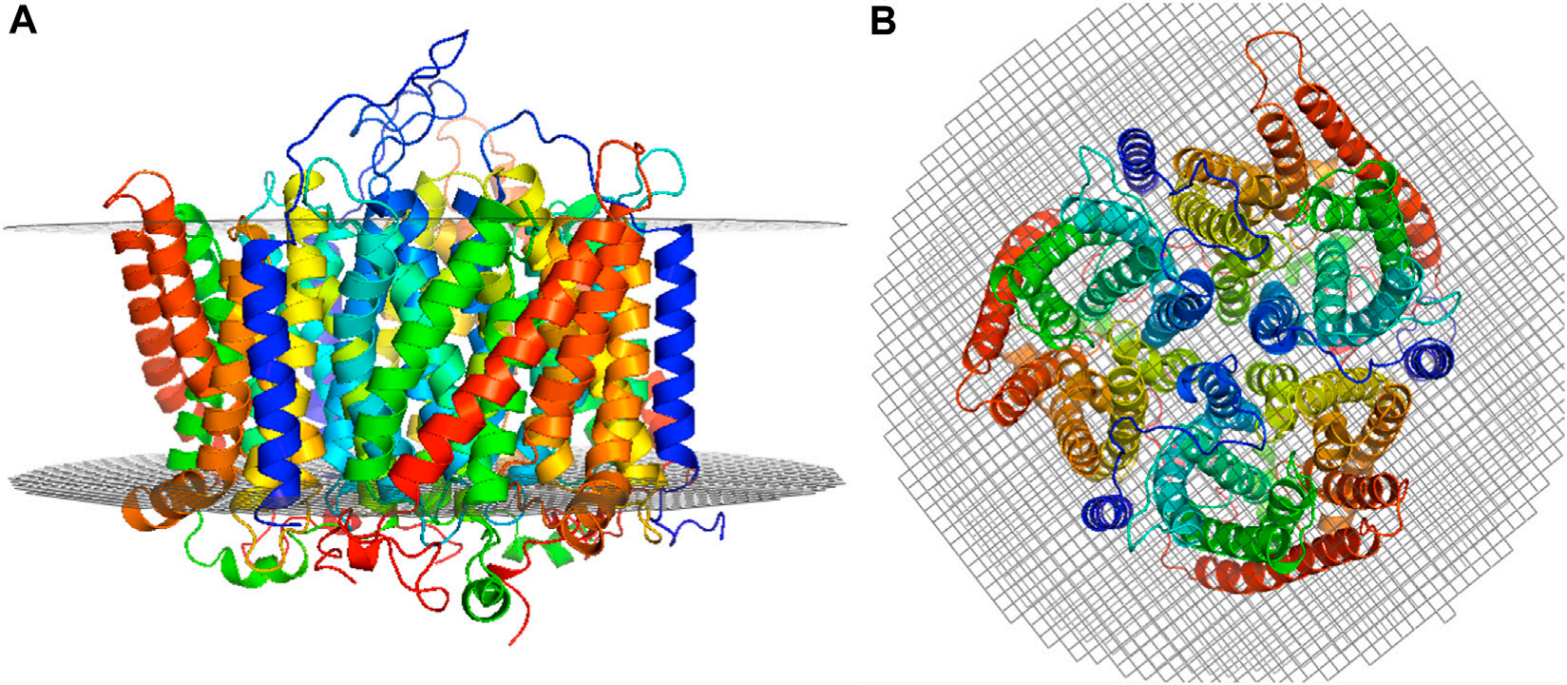
Selected RhD_1_RhAG_2_ trimer model. The RhD monomer is positioned as chain A (relative to the numbering of the structural template human RhCG, PDB ID: 3HD6) and the trimer positioned in a lipid bilayer using OREMPRO (Postic et al., 2016b). The extracellular compartment is at the top of figure. **(A)** Lateral view and **(B)** top view.

The best trimer for the RhD_1_RhAG_2_ with the RhD monomer positioned as chain A (relative to the numbering of the structural template human RhCG, PDB id: 3hd6, see Figure 1) is shown in Figure 2, positioned in a lipid bilayer using OREMPRO (Postic et al., 2016b).

### 3.2 Global flexibility of Rh trimers in molecular dynamics simulations

We have performed 1.5 µs of MD simulations for each of the trimer compositions: RhAG_3_, RhD_1_RhAG_2_ and RhD_2_RhAG_1_ (see Figure 1). In addition, 500ns of MD simulations for the non-physiological RhD_3_ system was done (see Figure 1). For all systems, the RMSD with respect to the initial conformation reached a plateau after 50 ns of simulation, indicating stabilisation of the protein structure (see Supplementary Figure S3).

**FIGURE 3:**
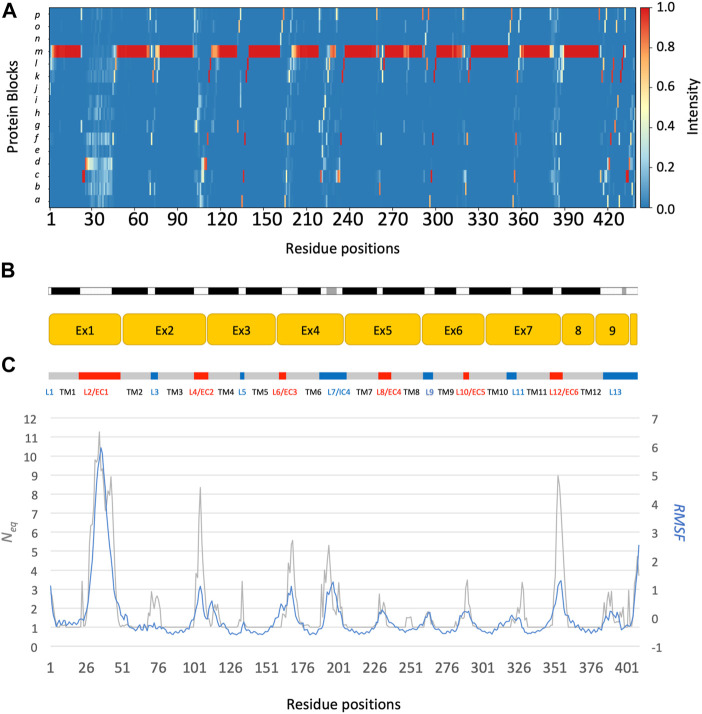
RhAG protein. The residues range from 1 to 409, from left to right. **(A)** Protein Blocks (de Brevern et al., 2000)assignment for the analysis of local conformations, extracted from each snapshot of the molecular dynamics simulations using the PBxplore tool (Barnoud et al., 2017). **(B)** Stride secondary structure assignment (Frishman and Argos, 1995), with α-helices in black, and 3_10_ helices in grey; Limits of the 10 exons of the protein; Position relative to the lipid bilayer, with intracellular domains (IC) in blue, transmembrane (TM) domains in grey, and extracellular domains in red (EC). **(C)** *N*_*eq*_, for equivalent number of Protein Blocks, in grey (with the axis to the left) and average, normalized RMSF, in blue (with the axis to the right).

**FIGURE 4:**
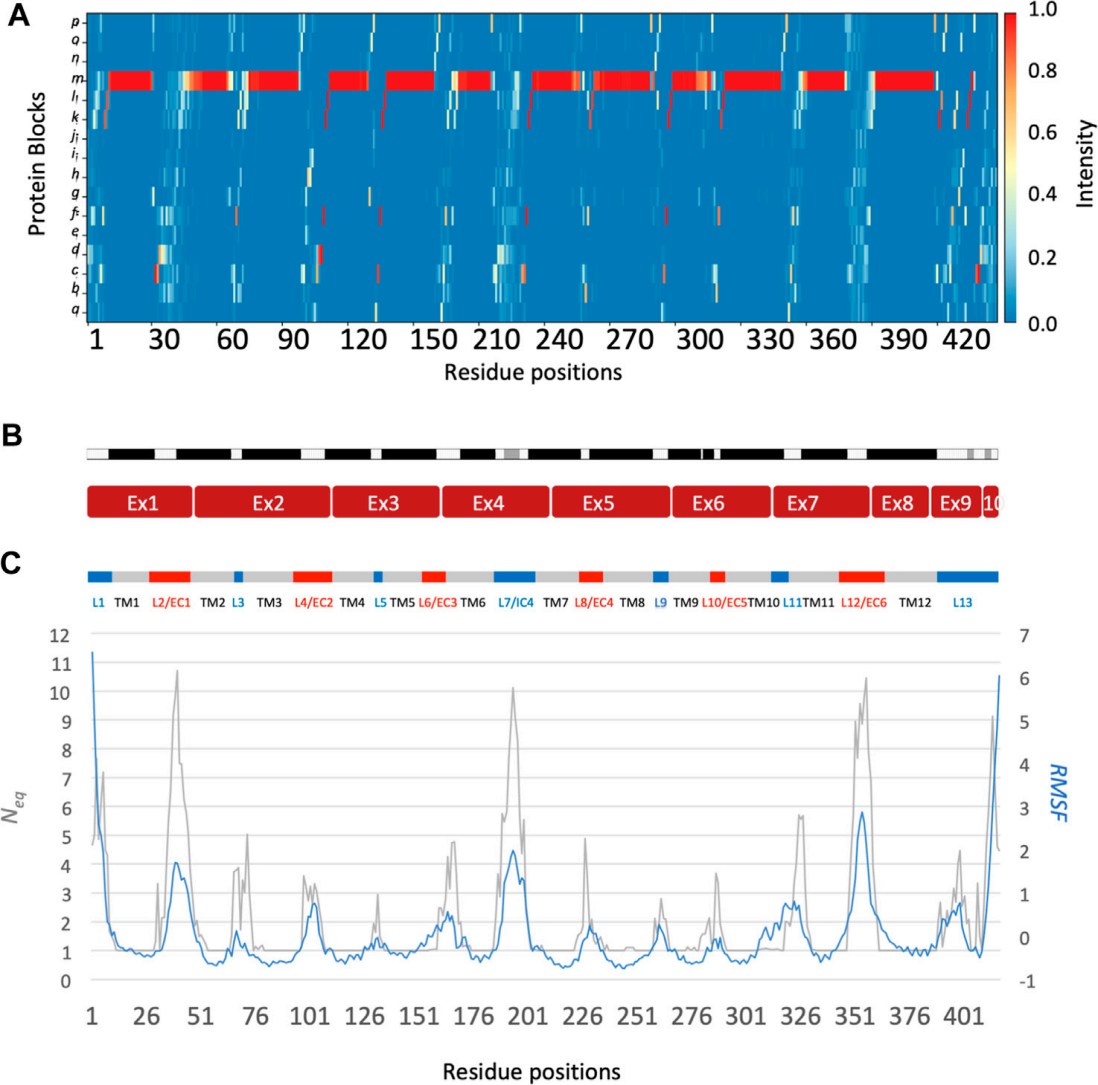
RhD protein. The residues range from 1 to 417, from left to right. See Figure 3 for legend.

**FIGURE 5:**
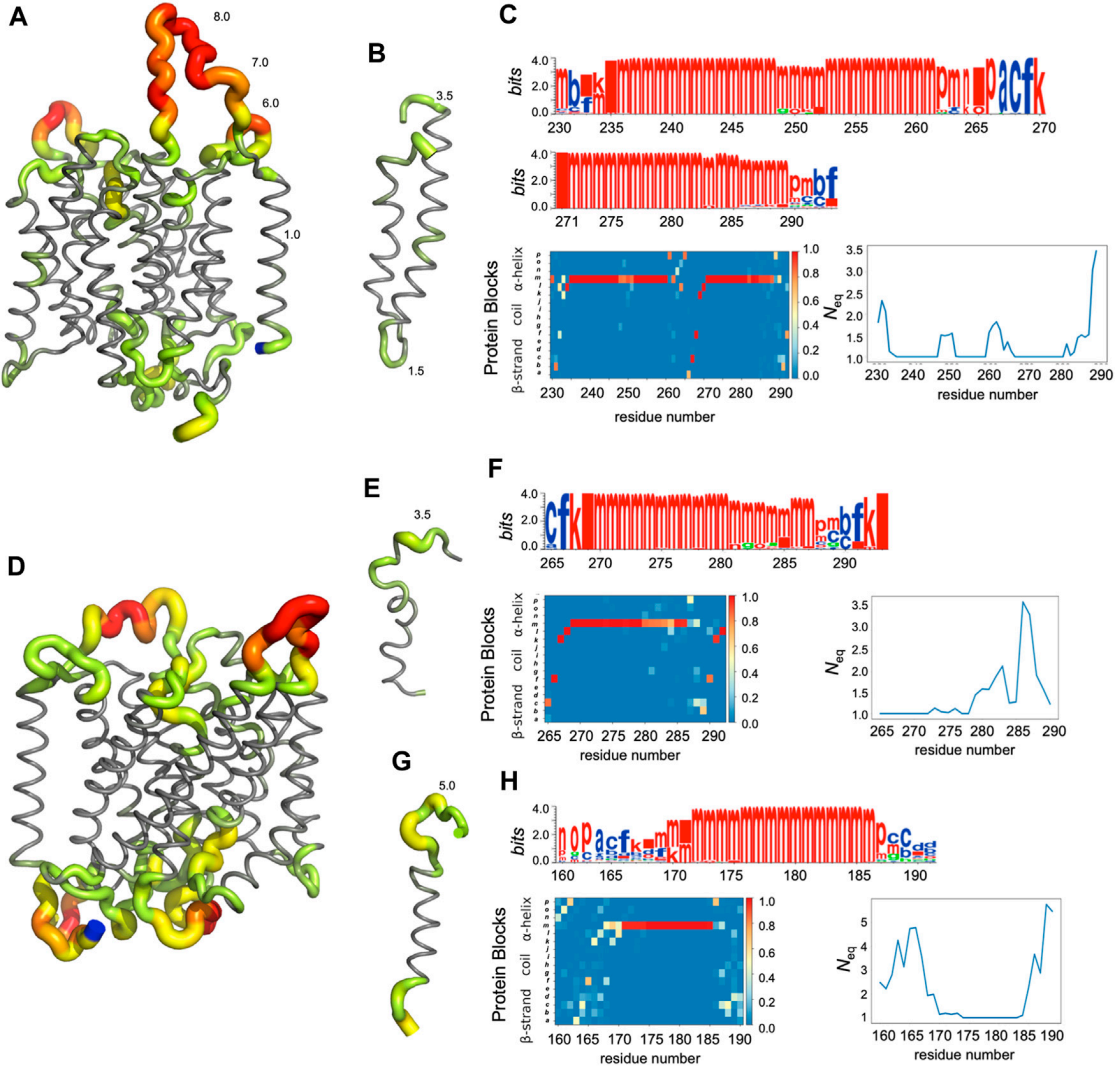
Analysis of some regions of RhAG **(A–C)** and RhD **(D–H)**. **(A)** Global view of RhAG coloured with *N*_eq_ values,* **(B)** zoom on helices 8 and 9 (positions 230-290), **(D)** global view of RhD coloured with *N*_eq_ values,* **(E)** zoom on helix 9 (positions 265-292) and **(G)** on helix 6 (positions 160-190), **(C) (F)** and **(H)** are the PB analyses (with logo, PB map and *N*_eq_). Blue: C-terminal residue. *N_eq_ gradient: from grey (*N*_eq_ = 1.0), trough green (2.0 > *N*_eq_ > 3.0), yellow (4.0 > *N*_eq_ > 5.0) and orange (6.0 > *N*_eq_ > 7.0), to red (*N*_eq_>8.0).

The main behaviours of RhAG and RhD are summarized in Figures 3, 4. The analysis with Protein Blocks (PB) (de Brevern et al., 2000; Barnoud et al., 2017) reveals that most transmembrane domains of both RhD and RhAG (in Figures 3B, 4B) are α-helices, associated to PB *m* (see Figures 3A, 4A). They are highly rigid as their *N*_eq_ values are equal to 1.0 (Akhila et al., 2020) (see Figures 3C, 4C), i.e., only one PB is observed during the whole simulation. Nonetheless, we observe local variations in several helices, especially in the middle of helices 8 and 9 in RhAG (see Figures 3B, C) and in helix 9 in RhD (see Figures 4E, F). For these residues, *N*_eq_ reaches 2.0. This value remains relatively low because the conformation remains predominantly helical. In Figure 5, we show several examples of different conformations illustrating the PB transitions in these regions. Increased conformation variability is also observed at the extremities of several helices, with a higher *N*_*eq*_ (see Figures 3C, 4C, and the orange and yellow colours in Figures 3A, 4A). This applies in particular to the N-terminal ends of helices 2, 3, 6, 11 and 12 of both RhAG and RhD, often associated with the PB series *cklm,* with a reasonably low *N*_eq_ of 2.0*.* Similarly, variations are observed at the C-terminal extremities of helices 2, 3, 7, 8, 9 and 11 of RhAG and helices 2, 3, 6, 7, 9 and 11 of RhD, often associated to the PB series *mop* (see Figures 3A, 4A). These variations of helical extremities are mainly directed by connecting loops (de Brevern et al., 2002).

### 3.3 Conformational behaviour of the most mobile protein regions

In fact, loop analysis shows which loops are disordered, flexible, or relatively rigid (Akhila et al., 2020). In both proteins, 3 loops are disordered, with a *N*_eq_ of 8.0 or more: loops 2, 4 and 12 in RhAG (the first, second and last extracellular loops) and loops 2, 7 and 12 in RhD (respectively the first extracellular loop, the fourth intracellular loop and the last extracellular loop, respectively). Several loops are not disordered but can be considered flexible, with an *N*_eq_ between 4.0 and 8.0: loops 6 and 7 in RhAG (the third extracellular and fourth intracellular loops), and loops 3, 6, 8 and 10 (respectively the second intracellular, third extracellular, fourth extracellular and sixth intracellular loops, respectively). The more rigid loops, with an *N*_eq_ less than 4.0 were: loops 3, 5, 8, 9, 10 and 11 in RhAG (all very small, according to Stride’s (Frishman and Argos, 1995) secondary structure assignment), and loops 4 and 8 in RhD (both small intracellular loops). Within the loops, several short alignments of PBs or structural words (de Brevern et al., 2002) show a β-sheet conformation: loops 2 and 4 in both RhAG (near residues 26 and 107) and in RhD (near residues 32 and 105). Most interestingly, the seventh loop of RhAG has a well-defined helical structure around residue 180. This loop is the fourth intracellular loop and the persistence of such local conformations may play a role in protein function.

### 3.4 Comparison of the different flexibility/disorder descriptors for protein dynamics analysis

The correlation between normalized B-factor values and normalized RMSF in both RhD and RhAG was high (Pearson’s coefficient *r* = 0.85 for RhAG loops, *r* = 0.83 for RhD loops). The obtained correlation is higher than previously observed in datasets of varied, representative globular proteins (Bornot et al., 2011; Narwani et al., 2018). However, some differences between RMSF and *N*_eq_ were observed. It should be recalled that the RMSF reflects a “global” structural variation, measuring the difference of all protein conformations with respect to a reference average conformation, whereas the *N*_eq_ is a local quantification, describing the protein backbone conformation based on the dihedral angles of 5 consecutive residues, i.e., PBs. It is therefore possible to have an *N*_eq_ of 1 (ultra-rigid) associated with a very high RMSF, i.e., a mobile region surrounded by deformable regions. The opposite (a low RMSF with a high *N*_eq_) is observed less frequently and represents strong local variations/oscillations of a protein region located in a globally more stable environment. RhAG loops 4 and 12 (the second and sixth extracellular loops) are the most prominent examples of this. In RhD, the four loops previously mentioned as flexible (loops 3, 6, 8 and 10) had a low RMSF compared to their high *N*_eq_.

### 3.5 Influence of the trimer composition on protein dynamics

The differences between the systems depending on the trimer composition were also analysed. The normalized RMSF for either RhAG or RhD showed only slight differences as a function of the trimer composition (see Supplementary Figure S4). A slightly lower RMSF was observed in RhAG for trimers with 3 RhAG subunits around residues 193 to 201 (see Supplementary Figure S4A), which are part of the large fourth intracellular loop (seventh loop). A very small increase in RMSF was observed in RhD for trimers with 3 RhD subunits around residues 225 to 237, which is the fourth extracellular loop (eighth loop, see Supplementary Figure S4A). It is quite remarkable to see such comparability in a system of this size.

**FIGURE 6:**
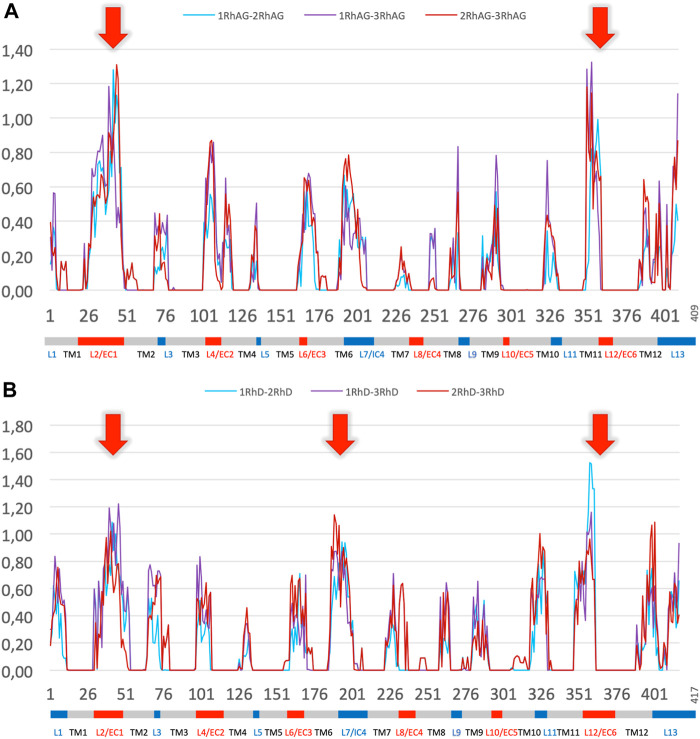
∆PB for the different trimer compositions for RhAG and RhD. Panel **(A)** RhAG. Blue: ∆PB between trimers with 1 RhAG subunit and trimers with 2 RhAG subunits; Purple: ∆PB between trimers with 1 RhAG subunit and 3 RhAG subunits; Red: ∆PB between trimers with 2 RhAG subunits and 3 RhAG subunits. Red arrows indicate the highest ∆PB for RhAG, in loops 2 and 12. Panel **(B)** RhD. Blue: ∆PB between trimers with 1 RhD subunit and trimers with 2 RhD subunits; Purple: ∆PB between trimers with 1 RhD subunit and 3 RhD subunits; Red: ∆PB between trimers with 2 RhD subunits and 3 RhD subunits. Red arrows indicate the highest ∆PB for RhD, in loops 2, 7 and 12 for RhD.

To further explore the variations in local conformations for RhAG and RhD, we calculated ΔPB to compare the PB frequencies observed for the systems with different RhAG and RhD composition. Figure 6A shows ΔPB between complexes with 1, 2 or 3 RhAG and Figure 6B shows similar information for systems with 1, 2 or 3 RhD. The comparison of the global distributions of PBs between the different systems does not reveal any particularities, i.e., no system had a region with very different conformational sampling. Outside of the disordered areas, there are no differences in ΔPB greater than 0.2. Strikingly, the trimer composition has no significant effect on the sampling of local protein conformations. This result may seem surprising, even counterintuitive. It should be clearly understood that the behaviour of a transmembrane protein complex constrained in a lipid environment is less labile than a globular protein. We thus have variations which are in fact quite classic, but do not show any strong change between the different systems.

Significant variations in ΔPB were observed depending on the region of the protein. As expected, transmembrane regions with low *N*_eq_ calculated for individual chains also had low ΔPB. Most of the loops had a limited ΔPB of 0.6 or less, i.e., the difference was less than 1/3 of the sampling. The maximum variability (see red arrows in Figure 6 for ΔPB greater than 1.0) was observed in the largest, disordered loops (with a *N*_*eq*_ of 8.0 or more): loops 2, and 12 for RhAG (respectively the first and the sixth extracellular loops, respectively), and loops 2, 7 and 12 for RhD (respectively the first extracellular loop, the fourth intracellular loop and the sixth extracellular loop, respectively). The last disordered loop with a *N*_*eq*_ of 8.0 was the 4th loop of RhAG; it was also associated with a ΔPB at the upper end of the ΔPB values, at 0.8.

## 4 Discussion and conclusion

In this work, we have proposed state-of-the-art trimer models for the human RhAG and RhD proteins, of major importance in transfusion medicine, and performed substantial molecular dynamics simulations (over 3µs total) to sample the local conformations of the RhAG and RhD proteins.

All the trimer structural models used in the molecular dynamics study were built by homology, using the same RhCG structure as a template (Figure 1). This choice was justified by a preliminary estimation of the quality of the RhAG_3_ trimer models, obtained using different approaches. Indeed, in our previous study (de Brevern et al., 2018), the best monomer model for RhD was obtained with a multi-template approach based on the structural templates of RhCG (Gruswitz et al., 2010) and NeRH50 (Li et al., 2007; Lupo et al., 2007). However, among the models of RhAG_3_ trimer built using as a template NeRH50 or RhCG alone, or together in the multi-template approach, the best structural models were obtained using RhCG alone as a template according to both DOPE (Shen and Sali, 2006) and HPM (de Brevern and Hazout, 2003; Esque et al., 2015; Téletchéa et al., 2023) scores (Supplementary Figure S5). The high sequence identity of RhCG with both RhAG (52.32%) and RhD (31.65%) made this template appropriate to build all trimer models.

It is important to note that correct positioning in the membrane can be obtained only for complete trimer models. Attempts to insert RhD and RhAG monomers in the lipid bilayer using OPM (Lomize et al., 2006; Lomize et al., 2011) and OREMPRO (Postic et al., 2016b) result in a high variability and inconsistencies between the models (see Supplementary Figure S6A), clearly showing that the monomeric forms cannot exist in a membrane environment on their own. Conversely, trimers are positioned harmoniously in the bilayer (see Supplementary Figure S6B), further corroborating the necessity of modelling the whole trimer rather than only one subunit, for a relevant analysis of protein dynamics.

Two major events occurred during the course of this work. First, AlphaFold2 greatly advanced the field of molecular modelling (Jumper et al., 2021). On average, it has made it possible to obtain 25% more residues and to reach 10% of the additional human proteome (Tunyasuvunakool et al., 2021). However, it also has recognized limitations (Tourlet et al., 2023), particularly for transmembrane proteins (He et al., 2023; Karelina et al., 2023). The generation of an AlphaFold2 model to compare ours to was disappointing, with a lack of cohesion in the helical domain making insertion into a membrane, for example, inefficient. This underperformance of AlphaFold2 is consistent with what we have recently observed when modelling two other blood group proteins, BCAM and ERMAP (Floch et al., 2023a; Floch et al., 2023b). Very recently, the structure of RhCE complexed with RhAG was obtained by cryo-EM (Vallese et al., 2022). We built a model based on this new structure, using the methodology described above, and compared it to the model used in the present study. The RMS deviation was less than 0.2 Å on Cα, demonstrating the high reliability of the model we had previously built and were using, and allowing its use without reserve for future studies.

As underlined by a multiple studies, the AlphaFold2 model does not work very well for membrane proteins, and people are working on new AI-based approaches that could improve it in the future with already interesting results (Wang et al., 2022; Huang and Ecker, 2023). A high fraction of membrane proteins form oligomeric structures, however, by often predicting them as monomeric, AlphaFold2 offers models that are not very useable (Dobson et al., 2023). Several approaches are being developed to overcome these limitations and also discover possible multiple conformations, particularly for globular proteins (Silva et al., 2023). A major advantage of artificial intelligence approaches is their speed of execution (after the learning phase) and this could be a great advantage compared to molecular dynamics simulation approaches, which are very computationally expensive (Tian et al., 2021; Zhang et al., 2022).

During MD simulations, the RhD and RhAG monomers behaved in a similar manner regardless of the trimer composition. The variations that were observed between the trimers of different compositions were similar to the variations that could be observed between the replicates for the exact same system. The strength of our work is to have sampled a large number of conformations, by proposing several closely related models, i.e., with the odd monomer at different chain positions (A, B or C) within the trimer models. Interestingly, MD simulations of the non-physiological RhD_3_ homotrimer model did not show major differences with MD simulations of RhD with other trimer compositions. Careful analysis of the global and local conformational variability of the systems considered here demonstrated the stability of the developed models with respect to the trimer composition and to the exact position of the RhAG and RhD chains.

The proposed RhAG/RhD trimer models can be used to delve into the details of the dynamics and interactions of Rh systems and variants, even if we do not model all elements of the physiological system. Firstly, we did not consider the post-translational modifications (PTM) of the proteins. Residues 12 and 186 of RhD proteins are S-palmitoylated, and RhAG proteins, as indicated by their name, are glycosylated (Moore and Green, 1987; Hartel-Schenk and Agre, 1992; Anstee and Tanner, 1993). However, the introduction of these PTMs would significantly increase the complexity of the (already large) system and its analysis. The second limitation of our approach is that human Rh proteins are part of a larger protein complex (Nicolas et al., 2003), and the partner proteins were not modelled in this work. Nonetheless, none of the other proteins of the Rh complex are strictly required for the expression of the Rh antigens, as revealed by the persistence of the Rh proteins in individuals lacking the different proteins of the complex (Colin, 2002), and supported by experimental data (Mouro-Chanteloup et al., 2003).

The proposed models are of crucial importance for the investigation of the impact of genetic variants on the blood group antigens’ structure and function. The D antigen, carried by the RhD protein, is crucial in transfusion medicine, due to its high variability with more than 700 alleles reported to-date (Floch et al., 2021a) and to the clinical consequences of anti-D antibodies (Chou and Westhoff, 2010). Assessing the consequences of these genetic variants is a challenge in transfusion medicine. The limited resource of D-negative blood should be preserved for D-negative recipients and carriers of clinically relevant RhD genetic variants (susceptible to producing anti-D if exposed to the standard D antigen). This work is an important step towards a better understanding of the D antigen conformations and the identification of relevant epitopes. Experimental studies and international collaborative efforts have tested many monoclonal anti-D antibodies with RhD genetic variants to try to study the epitopes to the RhD protein (Cartron et al., 1996; Scott, 1996; Scott, 2002). These experimental studies have not considered the 3D conformations of epitopes and the interpretation of the experimental results remain limited.

Using our molecular dynamics simulations to study epitopes, and Rh genetic variants could be particularly interesting and shed light on various experimental findings. The positions of the two biggest extracellular loops (loops 2 and 12) of RhD within the model are consistent with experimental data: mutations in these loops alter the antigens significantly and are responsible for alloimmunisation (Cartron et al., 1996). Other residues are known to play a role in the expression of D antigen epitopes despite their location in transmembrane (TM) domains: residues 169, 170 and 172 in the 6^th^ TM domain (Rouillac et al., 1995), residue 238 is in the 8^th^ TM domain (Noizat-Pirenne et al., 2002), and residue 283 is in the 9^th^ TM domain (von Zabern et al., 2013). Mutations in TM domains could have a long-range effect on extracellular parts of the protein, indirectly modifying the protein’s epitopes. Molecular dynamics simulations of the numerous RhD and RhAG genetic variants (Chou and Westhoff, 2010) will be interesting to analyse for subtle conformational changes. In a close future, it would also be interesting to carry out simulations using coarse-grained approaches, which allow long simulation times to be achieved with relatively good reliability and are particularly well suited to transmembrane systems (Rao et al., 2020; Souza et al., 2021).

## Web resources

 

**Table:** 

Name	url
Clustal Omega	http://www.ebi.ac.uk/Tools/msa/clustalo/
ConSurf	https://consurf.tau.ac.il/
HMMTOP	http://www.enzim.hu/hmmtop/
HHpred	http://toolkit.tuebingen.mpg.de/hhpred
HPM	http://www.dsimb.inserm.fr/dsimb_tools/hpmscore/
MAIDEN	http://www.dsimb.inserm.fr/maiden/
MEMSAT	http://bioinf.cs.ucl.ac.uk/psipred/?memsatsvm=1
Modeller 9.12	https://salilab.org/modeller/
MolProbity	http://molprobity.biochem.duke.edu
OPM	http://opm.phar.umich.edu
OREMPRO	http://www.dsimb.inserm.fr/dsimb_tools/OREMPRO/
PBxplore	https://github.com/pierrepo/PBxplore
PDBePISA	https://www.ebi.ac.uk/pdbe/pisa/
Procheck	https://www.ebi.ac.uk/thornton-srv/software/PROCHECK/
ProFit 3.1	http://www.bioinf.org.uk/programs/profit/
Protein DataBank	http://www.rcsb.org/pdb/
Protter	http://wlab.ethz.ch/protter/start/
PSI-BLAST	http://blast.ncbi.nlm.nih.gov/
RCSB PDB	https://www.rcsb.org/
UniProt	http://www.uniprot.org/

## Data Availability

The raw data supporting the conclusions of this article will be made available by the authors, without undue reservation.
