# Supplementary material for: Molecular dynamics of the human RhD and RhAG blood group proteins
Source: Front Chem. 2024 Mar 19;12:1360392. doi: 10.3389/fchem.2024.1360392 (PMC10985258; doi:10.3389/fchem.2024.1360392)
Supplement: Supplementary file 1 [file DataSheet1.pdf]

Supplementary Material

```
#
#
#  Percent Identity Matrix - created by Clustal2.1
#
#
```

1: 3K3F	100.00	18.78	17.08	19.47	18.56	20.17	17.28	17.11	14.41
2: 1XQE	18.78	100.00	37.31	37.02	35.04	21.47	19.22	21.41	22.09
3: 2B2F	17.08	37.31	100.00	39.49	40.36	22.22	17.97	21.71	21.49
4: 5AEZ	19.47	37.02	39.49	100.00	60.91	20.82	14.86	21.12	19.71
5: 5AEX	18.56	35.04	40.36	60.91	100.00	20.17	14.79	21.71	18.84
6: 3B9W	20.17	21.47	22.22	20.82	20.17	100.00	24.86	33.25	35.75
7: RHD	17.28	19.22	17.97	14.86	14.79	24.86	100.00	31.65	35.70
8: 3HD6	17.11	21.41	21.71	21.12	21.71	33.25	31.65	100.00	52.32
9: RHAG	14.41	22.09	21.49	19.71	18.84	35.75	35.70	52.32	100.00

Supplementary Table 1. Sequence identity matrix with Clustal Omega webserver (1) for human RhAG (Uniprot Q02094), human RhD (Uniprot Q02161) (2), and potential structural templates with crystal structures for their homology modelling: urea transporter of *Desulfovibrio vulgaris* (PBD id 3K3F (3)), AmtB of *Escherichia coli* (PBD id 1U7G (4)), Amt-1 of *Archaeoglobus fulgidus* (PBD id 2B2F (5)), Mep2 of *Candida albicans* (PBD id 5AEZ (6)), Mep2 of *Saccharomyces cerevisiae* (pdb id 5AEX (6)), Rh50 from *Nitrosomonas europaea* (PDB id 3B9W (7)) and human kidney RhCG (pdb id 3HD6 (8)).

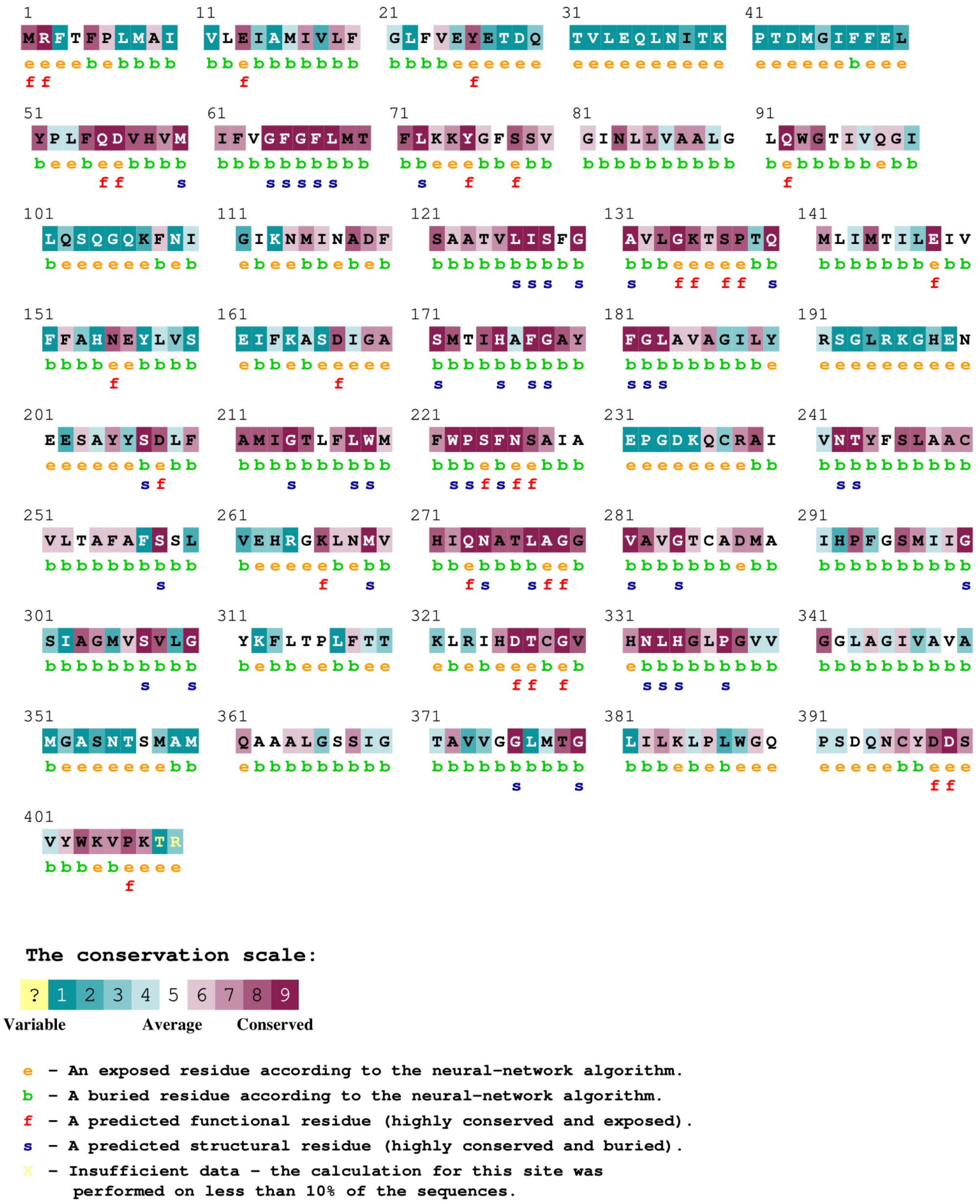

Supplementary Figure 1. Sequence conservation for RhAG protein with Consurf webserver (9, 10)(Accessed 28/08/2023), with default parameters.

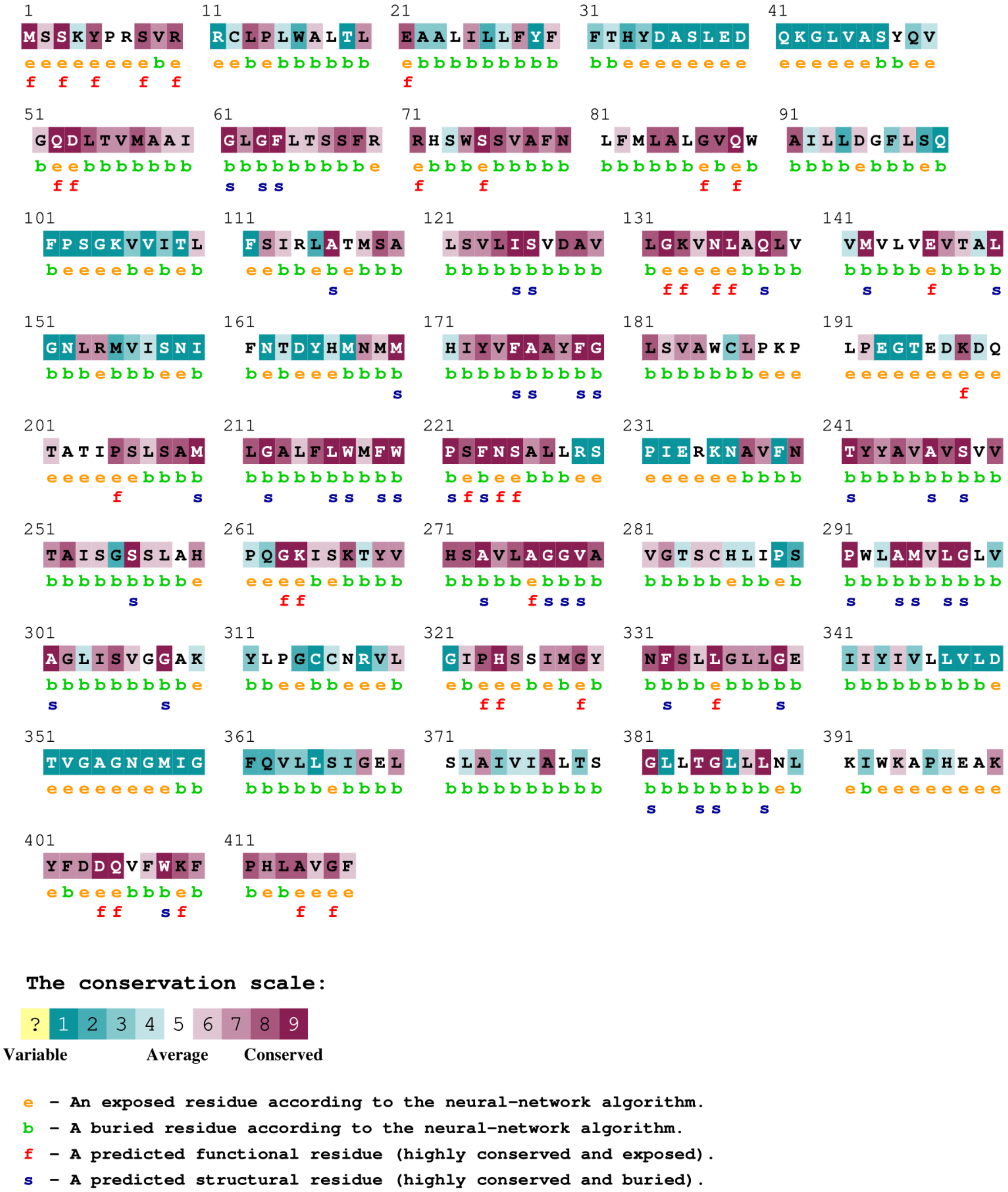

Supplementary Figure 2. Sequence conservation for RhAG protein with Consurf webserver (see Supplementary Figure 1 for details).

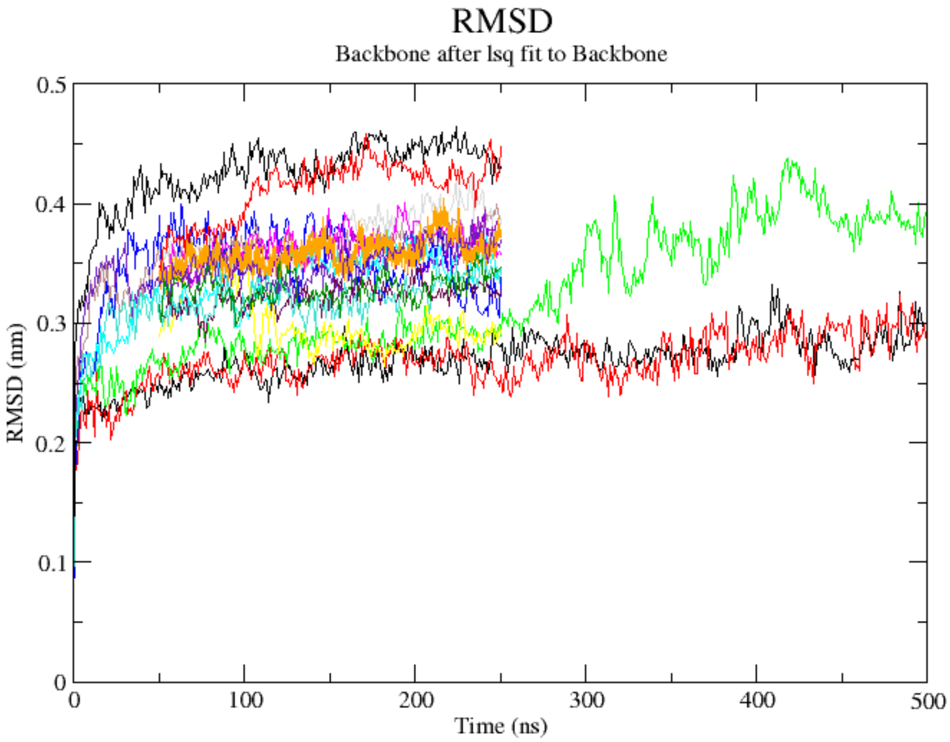

Supplementary Figure 3. Root Mean Square Deviation (RMSD) over all molecular dynamics simulations. Three trajectories were 500 ns long (three replicates for the $RhAG_3$ system, with red, green and black colours), the others were 250ns long (two replicates for each of the RhD_1RhAG_2 systems in blue, orange, cyan, dark green, pink and brow colours, each of the RhD_2RhAG_1 systems, in red, black, grey, magenta, purple and yellow colours, and for the RhD_3 system, in black and red colours). All trajectories reach a plateau before 50ns, so the first 50ns were removed for the analysis of local conformations.

A)

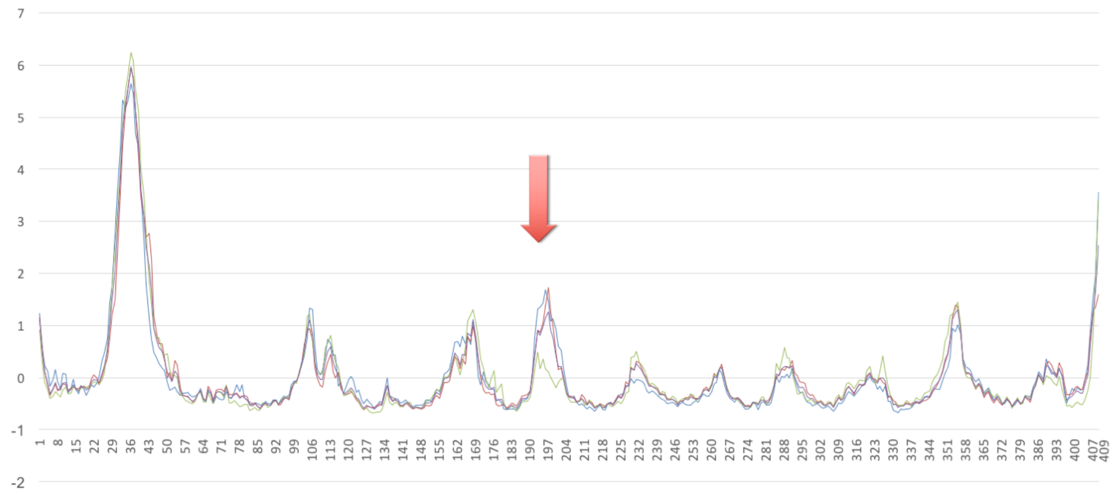

B)

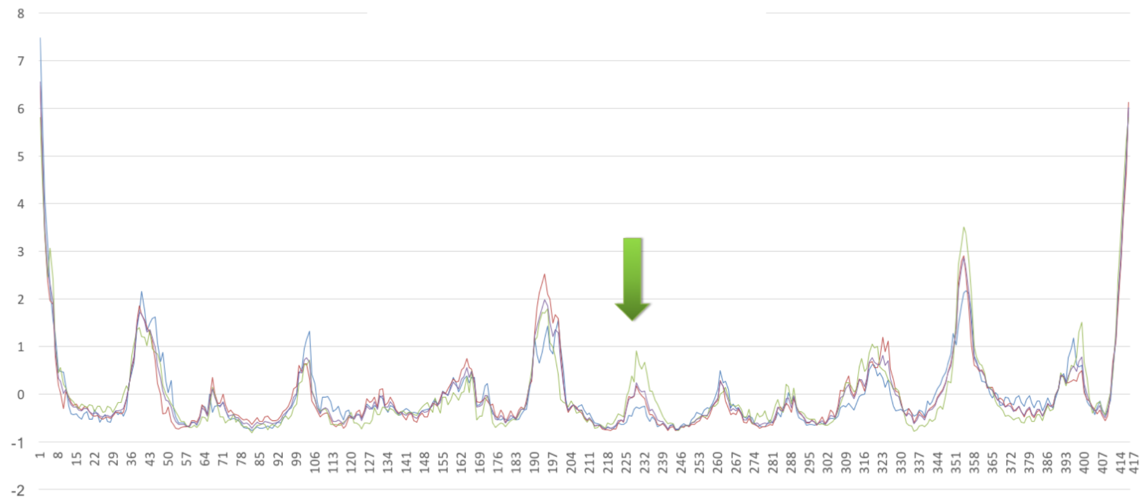

Supplementary Figure 4. Root Mean Square Fluctuation (RMSF) for molecular dynamics simulations: RhAG subunit (panel A) and RhD subunit (panel B), by trimer composition.

Blue: 1 monomer of either (A) RhAG or (B) RhD within the trimer; Red: 2 monomers of either (A) RhAG or (B) RhD within the trimer; Green: 3 monomers of either (A) RhAG of (B) RhD; Purple: mean RMSF for the three compositions (1, 2 or 3 monomers). On the horizontal axis: residue position; on the vertical axis: RMSF.

A slightly lower RMSF was observed in RhAG for trimers composed of 3 RhAG monomers around residues 193 to 201 (red arrow on panel A). A very small increase in RMSF was observed in RhD for trimers composed of 3 RhD monomers around residues 225 to 237, which is the 4th extracellular loop (8th loop, green arrow on panel B).

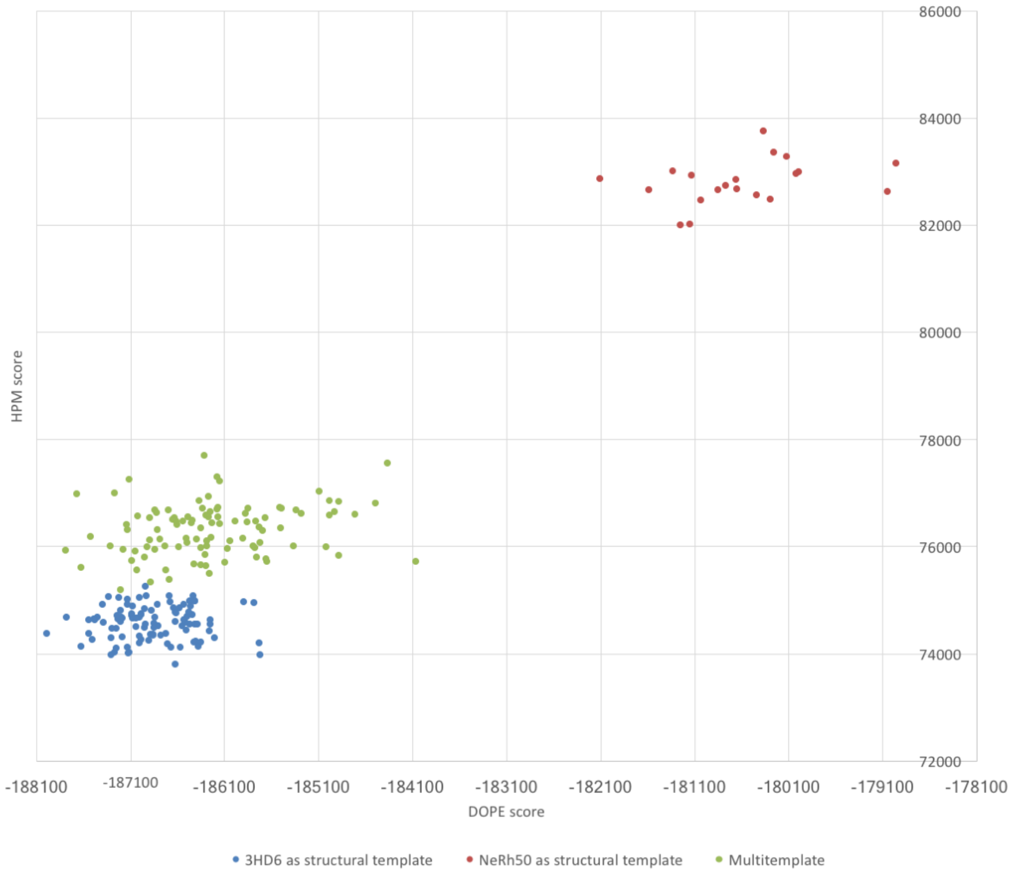

Supplementary Figure 5. Three series of models for $RhAG_3$ trimer from different structural templates, modelled in Modeller 9.12 (11, 12). Red: Nitrosomonas Europaeae Rh50 (PDB id 3B9W (7)) as a structural template; blue: human kidney RhCG (PBD id 3HD6 (8)) as a structural template; red: both structural templates combined in a multi-template approach.

The models were evaluated with the Discrete Optimized Protein Energy (DOPE) potential implemented in Modeller (13) and the Hybrid Protein Model (HPM) webserver (14, 15). Lower scores are indicative of a better model. The HPM approach is dedicated to transmembrane proteins. The models with only RhCG as a model scored as well as the multitemplate models with DOPE scoring, but scored better with HPM.

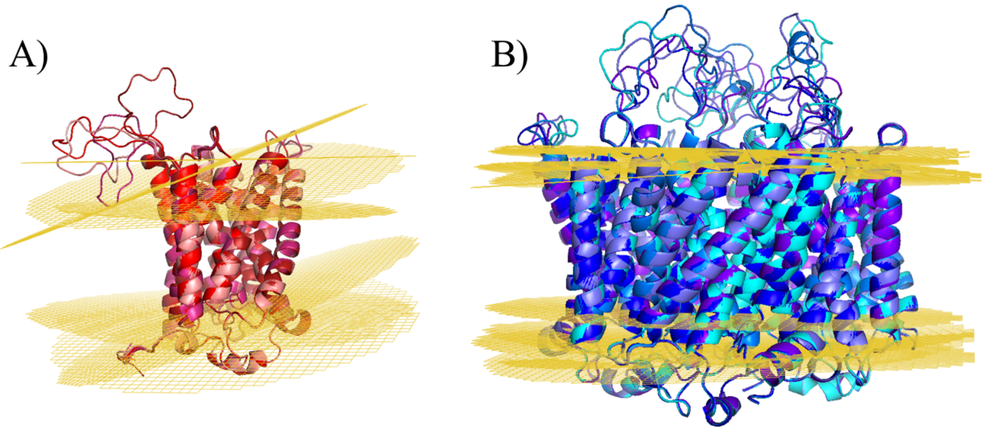

Supplementary Figure 6. Placement of RhAG proteins in a lipid bilayer with OREMPRO webserver (16). The extracellular compartment is at the topside of the figure. Panel A represents 3 different RhAG monomers and panel B, 3 different $RhAG_3$ trimers.

References

1. Sievers F, Wilm A, Dineen D, Gibson TJ, Karplus K, Li W, et al. Fast, scalable generation of high-quality protein multiple sequence alignments using Clustal Omega. Molecular systems biology. 2011;7:539.

2. Consortium U. UniProt: a hub for protein information. Nucleic acids research. 2015;43(Database issue):D204-12.

3. Levin EJ, Quick M, Zhou M. Crystal structure of a bacterial homologue of the kidney urea transporter. Nature. 2009;462(7274):757-61.

4. Khademi S, O'Connell J, 3rd, Remis J, Robles-Colmenares Y, Miercke LJ, Stroud RM. Mechanism of ammonia transport by Amt/MEP/Rh: structure of AmtB at 1.35 A. Science (New York, NY). 2004;305(5690):1587-94.

5. Andrade SL, Dickmanns A, Ficner R, Einsle O. Crystal structure of the archaeal ammonium transporter Amt-1 from Archaeoglobus fulgidus. Proceedings of the National Academy of Sciences of the United States of America. 2005;102(42):14994-9.

6. van den Berg B, Chembath A, Jefferies D, Basle A, Khalid S, Rutherford JC. Structural basis for Mep2 ammonium transceptor activation by phosphorylation. Nature communications. 2016;7:11337.

7. Lupo D, Li XD, Durand A, Tomizaki T, Cherif-Zahar B, Matassi G, et al. The 1.3-A resolution structure of Nitrosomonas europaea Rh50 and mechanistic implications for NH3 transport by Rhesus family proteins. Proceedings of the National Academy of Sciences of the United States of America. 2007;104(49):19303-8.

8. Gruswitz F, Chaudhary S, Ho JD, Schlessinger A, Pezeshki B, Ho CM, et al. Function of human Rh based on structure of RhCG at 2.1 A. Proceedings of the National Academy of Sciences of the United States of America. 2010;107(21):9638-43.

9. Ashkenazy H, Abadi S, Martz E, Chay O, Mayrose I, Pupko T, et al. ConSurf 2016: an improved methodology to estimate and visualize evolutionary conservation in macromolecules. Nucleic acids research. 2016;44(W1):W344-50.

10. Ashkenazy H, Erez E, Martz E, Pupko T, Ben-Tal N. ConSurf 2010: calculating evolutionary conservation in sequence and structure of proteins and nucleic acids. Nucleic acids research. 2010;38(Web Server issue):W529-33.

11. Sali A, Blundell TL. Comparative protein modelling by satisfaction of spatial restraints. Journal of molecular biology. 1993;234(3):779-815.

12. Martí-Renom MA, Stuart AC, Fiser A, Sánchez R, Melo F, Sali A. Comparative protein structure modeling of genes and genomes. Annual review of biophysics and biomolecular structure. 2000;29:291-325.

13. Shen MY, Sali A. Statistical potential for assessment and prediction of protein structures. Protein science : a publication of the Protein Society. 2006;15(11):2507-24.

14. Esque J, Urbain A, Etchebest C, de Brevern AG. Sequence-structure relationship study in all-α transmembrane proteins using an unsupervised learning approach. Amino acids. 2015;47(11):2303-22.

15. Téletchéa S, Esque J, Urbain A, Etchebest C, de Brevern AG. Evaluation of Transmembrane Protein Structural Models Using HPMScore. BioMedInformatics. 2023;3(2):306-26.

16. Postic G, Ghouzam Y, Gelly JC. OREMPRO web server: orientation and assessment of atomistic and coarse-grained structures of membrane proteins. Bioinformatics (Oxford, England). 2016;32(16):2548-50.
